# Magnesium treatment for neuroprotection in ischemic diseases of the brain

**DOI:** 10.1186/2040-7378-5-6

**Published:** 2013-04-25

**Authors:** Thomas Westermaier, Christian Stetter, Ekkehard Kunze, Nadine Willner, Furat Raslan, Giles H Vince, Ralf-Ingo Ernestus

**Affiliations:** 1 Department of Neurosurgery, University Hospital Würzburg, Josef-Schneider-Str. 11, Würzburg 97080, Germany; 2 Department of Neurosurgery, Klinikum Klagenfurt, Feschnigstraße 11, Klagenfurt am Wörthersee 9020, Austria

## Abstract

This article reviews experimental and clinical data on the use of magnesium as a neuroprotective agent in various conditions of cerebral ischemia. Whereas magnesium has shown neuroprotective properties in animal models of global and focal cerebral ischemia, this effect could not be reproduced in a large human stroke trial. These conflicting results may be explained by the timing of treatment. While treatment can be started before or early after ischemia in experimental studies, there is an inevitable delay of treatment in human stroke. Magnesium administration to women at risk for preterm birth has been investigated in several randomized controlled trials and was found to reduce the risk of neurological deficits for the premature infant. Postnatal administration of magnesium to babies after perinatal asphyxia has been studied in a number of controlled clinical trials. The results are promising but the trials have, so far, been underpowered. In aneurysmal subarachnoid hemorrhage (SAH), cerebral ischemia arises with the onset of delayed cerebral vasospasm several days after aneurysm rupture. Similar to perinatal asphyxia in impending preterm delivery, treatment can be started prior to ischemia. The results of clinical trials are conflicting. Several clinical trials did not show an additive effect of magnesium with nimodipine, another calcium antagonist which is routinely administered to SAH patients in many centers. Other trials found a protective effect after magnesium therapy. Thus, it may still be a promising substance in the treatment of secondary cerebral ischemia after aneurysmal SAH. Future prospects of magnesium therapy are discussed.

## Pathophysiology and molecular effects

The interruption of cerebral blood flow (CBF) is followed by a complex pathophysiological cascade which ultimately results in ischemic cell death. ATP-depletion and ischemic depolarization lead to excessive cellular calcium-entry. Under physiological conditions, calcium is an important second messenger whereas excessive calcium is toxic and induces mechanisms of cell destruction. Depending on the kind and severity of ischemia, tissue necrosis and/or apoptosis are the ultimate consequences [1]. Since the importance of calcium in ischemic cell death had been discovered, calcium antagonists have come into the focus of ischemic neuroprotection [2]. Magnesium has been called “nature’s physiologic calcium blocker” as it is a physiological mineral and interferes with calcium in a variety of ways [3]. The bivalent magnesium cation can compete with calcium ions for receptor binding or passage through ion channels. It dilates blood vessels by competitive inhibition of voltage-dependent calcium channels in vascular smooth muscle cells [4], improves rheological functions by inihibiton of platelet aggregation [5,6], and increases the deformability of red blood cells [7]. Under experimental conditions, it prevents cellular calcium influx and excitatory amino acid release in neurons by blockade of N-type and L-type calcium channels [8], prevents cellular calcium entry through NMDA-receptor channels [9], reduces calcium-induced mitochondrial dysfunction [10] and preserves cellular energy metabolism [11]. By these mechanisms, magnesium may inhibit or delay ischemic cell death during and after cerebral ischemic events.

## Physiological concentrations and side effects

**Figure 1:**
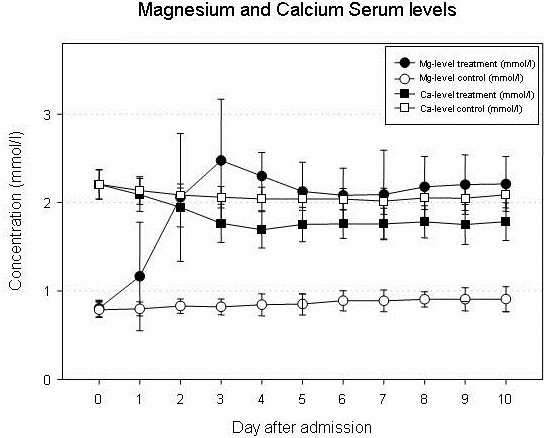
**Serum concentrations of magnesium and calcium.** After the start of intravenous magnesium administration, a significant decrease of the serum calcium concentration is observed.

In human serum, the physiological magnesium concentration is 0.7 – 1.1 mmol/l [12]. Magnesium is almost completely renally excreted. Oral intake may result in diarrhea but does not markedly increase serum concentrations unless patients suffer from renal insufficiency. Intravenous magnesium has been used for a long time in obstetrics for the treatment of eclampsia and in cardiology for the treatment of supraventricular tachycardia. Therefore, side effects are well-defined. During magnesium treatment, hypocalcemia develops (Figure 1). With increasing serum concentrations, hypotension and bradycardia occur and tendon reflexes disappear. Serum concentrations over 6 mmol/l can result in coma, respiratory insufficiency and cardiac arrest. In patients with renal failure, magnesium may quickly accumulate and result in hazardous side effects.

**Figure 2:**
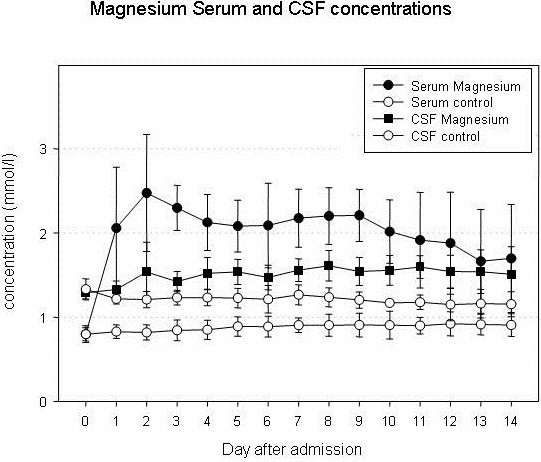
Even after prolonged intravenous magnesium administration, CSF concentrations remain distinctly lower than serum concentrations indicating a saturation of active transport via the blood brain barrier and blood-CSF-barrier.

Low blood pressure is a risk factor for aggravation of ischemic damage in states of cerebral ischemia reducing collateral flow in the ischemic penumbra. Therefore, magnesium doses must be kept low enough to ensure stable blood pressure if it is administered for the purpose of neuroprotection. In an experimental study of temporary middle cerebral artery occlusion (MCAO) in rats, serum concentrations of 2.0 – 2.5 mmol/l showed the highest neuroprotective effect [13]. In higher doses, the cardiodepressive effect seemed to limit the extent of neuroprotection. In the clinical use in stroke and SAH, reports about side effects are diverse. Most authors did not find significant side effects apart from hypocalcemia, occasional flushing or a slight tendency to bradycardia [14-20], others reported significant hypotension and cardiorespiratory depression in spite of close surveillance of serum levels [21]. Co-treatment with other pharmaceutics with a antihypertensive potential may be a possible factor. The doses that are required to maintain certain target levels vary widely and depend on renal excretion [19]. Serum electrolyte concentrations are to be followed frequently with consequent adaptation of infusion rates. With a mean of 1.27 ± 0.15 mmol/l, physiological cerebrospinal fluid (CSF) concentrations are higher than serum concentrations suggesting an active transport through the blood–brain-barrier and blood-CSF-barrier. However, CSF concentrations increase only moderately even after several days of high-dose administration (1.50 ± 0.16 mmol/l at serum levels of 2.14 ± 0.21) indicating a saturation of transport capacity [19] (Figure 2).

## Experimental data

The use of magnesium as a neuroprotective agent has been examined in various experimental models of cerebral ischemia with rather conflicting results. This may be explained by the variety of experimental models, the wide range of target parameters, and the different dosages, timing and routes of administration. Experimental data was collected by a Medline search for “magnesium”, “experimental” and “cerebral ischemia” or “cerebral hypoxia”.

### Global ischemia

Blair et al. did not find a reduction of hippocampal damage by a 5 mmol/kg intravenous bolus of magnesium chloride (MgCl_2_) immediately before 10 minutes of bilateral carotid occlusion in rats. Regarding the well-known concentration-related spectrum of side effects, it is remarkable that the authors did not observe any major side effects – especially regarding cardiovascular parameters - although the single intravenous bolus injection led to a tenfold elevation of plasma levels 10-fold [12,22]. The animals’ heart-rate, the most sensitive parameter during magnesium treatment, however, was not measured so that a cardiodepressive effect cannot be finally excluded [19].

Studies with slower resorption of magnesium, in contrast, showed neuroprotective effects in global ischemia. The subcutaneous administration of the same amount (5 mmol/kg) of magnesium sulfate (MgSO_4_) 48 hours before a 15-minute period of forebrain ischemia in rats resulted in a 24% and 39% reduction of cell damage in the hippocampal CA1- and CA3-area, respectively [23]. Zhou et al. found a 50% reduction of hippocampal damage by an intraperitoneal administration of 16 mmol/kg MgSO_4_ 30 minutes prior to 10 minutes of bilateral carotid artery occlusion [24]. However, neither body nor brain temperature were measured and regulated in these studies. In a series of studies using 8 minutes of bilateral carotid occlusion in rats, Zhu and coworkers found that a postischemic temperature drop could be the decisive factor for the neuroprotective effect of magnesium. When animals were kept strictly normothermic, magnesium did not reduce hippocampal damage. If body temperature was mildly reduced, hippocampal damage was significantly reduced after magnesium treatment compared to hypothermic controls [25,26].

### Permanent focal ischemia

Several groups investigated the protective efficacy in studies of permanent focal ischemia [27-30]. Apart from Roffe et al. [30], all authors reported reduced infarct volumes. In all of these studies, magnesium was administered either before or shortly after the onset of permanent middle cerebral artery occlusion (MCAO). Yang et al. evaluated the effect of magnesium administered 2, 6 or 8 hours after permanent MCAO. While the survival rate improved only in the 2-hour group, infarct volume decreased in the 2- and 6 hour groups and neurological outcome improved in all treatment groups [31]. This finding gave rise to the expectation that even delayed magnesium treatment might be effective in stroke patients.

### Temporary focal ischemia

In a rat model of 90 minutes of MCAO, Marinov et al. found a nearly 60% reduction of infarct volume after 24 hours by a preischemic intraarterial bolus of 90 mg/kg MgSO_4_. The authors concluded that this superior protective effect was due to the intraarterial route of administration [32]. However, this overwhelming neuroprotective effect could not be reproduced in a direct comparison of intraarterial and intravenous administration of the same dose in the same model [33]. The infarct volume – assessed after 7 days - was reduced by 25% compared to saline treated animals in both the intraarteial and the intravenous groups. The difference between assessment of infarct volume after 1 day and after 7 days rather suggests that the neuroprotective effect of magnesium may, in part, be temporary [33]. The results of the latter study were rather consistent with previous results of the same group [34].

In most studies, the neuroprotective effect of magnesium in transient focal ischemia was moderate but reproducible. Magnesium has shown synergistic effects with other substances like radical scavengers and immunosuppressants, which makes it an interesting substance for the development of combination therapies [27,34]. An experimental dose-finding study in transient MCAO in rats showed the highest neuroprotective effect in animals treated by a continuous elevation of serum levels to 2.0 – 2.5 mmol/l. Higher levels led to a cardiodepressive effect, probably limiting the neuroprotective efficacy [13].

## Hypoxia/Ischemia

Several authors have studied the neuroprotective potential of magnesium in perinatal asphyxia in animal models. In an study of hypoxia/ischemia, Spandou and coworkers subjected newborn rats to permanent unilateral carotid occlusion and 1 or 2 hours of hypoxia by reduction of the inspired oxygen fraction to 8%. Treatment with 4 doses of 500 mg/kg MgSO_4_ resulted in a delay of energy depletion and a reduction of hippocampal damage compared to saline treated control animals [35]. Using the same species and experimental model, Thordstein et al. reported a neuroprotective effect of MgSO_4_ in combination with the oxygen scavengers L-Methionine and Mannitol determined as a less pronounced reduction of hemispheric weight than in placebo treated control anaimals [36].

In contrast, magnesium treatment did not show a neuroprotective effect in a model of hypoxia/ischemia in piglets [37,38], where the animals were subjected to temporary bilateral carotid occlusion and a reduction of the inspiratory oxygen fraction to 0.12 until the ratio of phosphocratinine and inorganic phosphate, determined by magnetic resonance spectroscopy, had fallen to zero. The authors neither found a delay of energy depletion after MgSO_4_ treatment (400 mg/kg after 1 hour of resuscitation and 200 mg/kg after 24 and 48 hours) [38] nor a reduction of brain tissue damage [37]. In a model of 60 minutes of hypoxia without carotid ligation (inspiratory oxygen fraction 5 – 7%), in contrast, treatment with a bolus of 600 mg/kg MgSO_4_ followed by an infusion of 300 mg/kg through 1 hour of hypoxia did preserve cerebral energy stores in newborn piglets [39].

Experimental models of permanent and transient focal cerebral ischemia find their clinical counterparts in embolic stroke, events of temporary ischemia during neurosurgical operations or delayed vasospasm after subarachnoid hemorrhage, respectively, models of hypoxia/ischemia in the clinical situation of perinatal asphyxia. Thus, a series of clinical studies has been performed during the last 15 years evaluating the neuroprotective potential of magnesium after embolic stroke, its ability to prevent ischemic deficits in secondary vasospasm after aneurysmal SAH and its potential to ameliorate ischemic/hypoxic damage after perinatal asphyxia.

## Clinical trials

Clinical data was collected by a MEDLINE-search for “magnesium”, “clinical trial” and “stroke”, “subarachnoid hemorrhage” or “asphyxia”.

## Stroke

Promising results in experimental studies have raised high expectations that magnesium might be a valuable drug for the treatment of stroke patients. In 1984, a first clinical trial was launched investigating the tolerability of magnesium in human stroke [40]. In 1995, a randomized placebo-controlled pilot study was published [41], in which 30 patients were treated by a bolus infusion of 8 mmol MgSO_4_ followed by a continuous infusion of 65 mmol over 24 hours in order to double the physiological serum concentrations. 30 patients served as a control group. In the magnesium group, 30% of the patients were dead or disabled compared to 40% in the control group. The dose regimen had been chosen in accordance with previous studies on myocardial infarction [42] and because serum levels of approximately 1.5 mmol/l had shown a neuroprotective effect in experimental studies [32]. The initial bolus of 8 mmol, however, did not markedly elevate serum magnesium levels and target levels were only reached after 24 hours. A “dose optimization study” was conducted in 25 patients preparing a large clinical trial. Target levels were most rapidly reached by an initial bolus of 16 mmol MgSO_4_, still without cardiovascular side effects [16]. The IMAGES-trial (Intravenous Magnesium Efficacy in Stroke Trial) was launched in 1997 treating patients with ischemic stroke within 12 hours after the onset of symptoms and included 2598 patients [43]. After promising results of animal studies and small pilot trials, this large multicenter trial did not show an overall improvement of clinical outcome by magnesium treatment. Only a subgroup of patients with lacunar stroke profited from magnesium treatment [44] (Table 1).

**Table 1:** Clinical studies assessing the effects of intravenous magnesium in ischemic stroke

**Author**	**Study setup**	**Treatment arms and comedication**	**Individuals**	**Results**
Wester et al., 1984 [40]	Tolerability study	MgSO_4_ (15 mmol + 96 mmol/24h for 5 days)	14 patients with ischemic stroke	Magnesium-treatment well tolerated. No information about outcome
Muir et al., 1995 [41]	Randomized, placebo-controlled	MgSO_4_ (8 mmol + 65 mmol/kg for 24 hours) vs. placebo	60 patients with ischemic stroke	Magnesium-treatment well tolerated, tendency to better neurological outcome
Muir et al., 1998 [16]	Randomized, placebo-controlled	MgSO_4_ (8, 12 or 16 mmol + 65 mmol/kg for 24 hours) vs. placebo	25 patients with ischemic stroke	All dose-regimens well tolerated. No difference in outcome
The IMAGES investigators, 2004 [43]	Randomized, placebo-controlled	MgSO_4_ (16 mmol + 65 mmol/kg for 24 hours) vs. placebo	2589 patients with ischemic stroke	No over all reduction of death or disability (primary end-point), trend to benefit in lacunar stroke

After promising experimental data and a trend to better outcome, a large multicenter clinical trial failed to demonstrate a beneficial effect of intravenous magnesium in the over all study population. Solely the subgroup with lacunar infarction showed a tendency to better recovery after magnesium medication.

It has been thoroughly discussed why the IMAGES trial failed to reproduce the positive results gained in experimental studies. The sample size might have still been too small or side effects might have been hazardous in some patients masking a positive effect in the majority. Moreover, the mean delay from the onset of clinical symptoms to the beginning of treatment was 7 hours. Since the efficacy of neuroprotective agents may decrease the longer their administration is delayed [31], this issue is given consideration to by the currently conducted FAST-MAG trial (“Field Administration of Stroke Therapy – Magnesium”) in which patients are enrolled within the first 2 hours after the onset of stroke symptoms [45]. One drawback of the IMAGES trial may have been the lack of proper preclinical testing of the dose-regimen and study-design before entering a large multicenter trial. It was proposed that a target serum concentration twice as high as the physiological concentration may be the optimum treatment for stroke patients [16,41]. However, neither a preclinical dose-finding study using an established experimental stroke model nor a clinical (phase 2) dose-finding study had been conducted in advance to this large clinical trial.

## Subarachnoid hemorrhage

Most cases of spontaneous SAH are caused by the rupture of an intracranial aneurysm. Aneurysmal SAH is followed by a sequence of different forms of cerebral ischemia: First, global ischemia arises due to an elevation of intracranial pressure (ICP). Starting immediately after SAH, a dissociation of cerebral perfusion pressure and CBF has been found which enhances and prolongs the early perfusion deficit after SAH. It is likely to be caused by an acute vasoconstriction, a disseminated cerebrovascular reaction [46,47]. Finally, delayed vasospasm of subarachnoid vessels appears in a high percentage of SAH patients within the first 2 weeks after SAH. Subarachnoid vessels become increasingly spastic and cerebral blood flow (CBF) may fall below ischemic thresholds [48]. This feared complication affects 20 – 30% of SAH patients and may result in cerebral infarction, permanent morbidity or death. Delayed cerebral vasospasm after SAH is self-limiting after several days and, therefore, resembles a form of transient focal ischemia. This bears the opportunity to undertake neuroprotective measures prior to the onset of ischemia. In an animal model of temporary MCAO, a dose-finding study has demonstrated that the intra- and postischemic maintainance of serum concentrations between 2.0 and 2.5 mmol/l offered the highest neuroprotection [13].

The potential role for a treatment with magnesium was supported by the observation that hypomagnesemia is frequently found in SAH patients and correlates with the amount of blood in the subarachnoid space and with the patient’s neurological condition at the time of hospital admission. Hypomagnesemia arising during the course of treatment, in turn, correlates with the appearance of secondary neurological deficits and ischemic infarctions [49]. A series of clinical trials has been launched to assess the ability to reduce secondary neurological deficits after SAH (Table 2).

**Table 2:** Randomized clinical studies investigating the therapeutic effect of intravenous magnesium to prevent delayed vasospasm and secondary ischemic events and to improve outcome after aneurysmal subarachnoid hemorrhage

**Author**	**Study setup**	**Treatment arms and comedication**	**Individuals**	**Results**
Luo et al., 1996 [50]	Randomized, patient-blinded	MgSO_4_ (approx. 100 – 200 mmol per day for 2 – 3 weeks) vs. placebo	52 patients	Significant reduction of secondary neurological deterioration, reduction of delayed cerebral infarction
Veyna et al., 2002 [18]	Randomized, patient-blinded	Nimodipine vs. nimodipine + MgSO_4_ (25 mmol + 192 mmol/day for 10 days)	36 patients	Safe use of magnesium. Non-significant trend to improved clinical outcome
Van den Bergh et al., 2005 [17]	Randomized, double-blinded	Nimodipine vs. nimodipine + MgSO_4_ (64 mmol/day for 14 days)	283 patients	Reduction of delayed cerebral ischemia and trend to better neurological outcome
Schmid-Elsaesser et al., 2007 [51]	Randomized, double-blinded	Nimodipine vs. MgSO_4_ (10mg/kg + 30mg/kg/day for 7 days)	104 patients	No significant difference between magnesium and nimodipine
Muroi et al., 2008 [21]	Randomized, patient-blinded	Nimodipine vs. nimodipine + MgSO_4_ (16 mmol + 64 mmol/24h, maximum serum concentration 2.0 mmil/l)	58 patients	Trend to better clinical outcome after 3 and 12 months. Treatment was stopped in 16 patients due to hypotension, arrhythmias, respiratory arrest and myocardial infarction
Wong et al., 2010 [20]	Randomized, double blinded	Nimodipine vs. nimodipine + MgSO_4_ (20 mmol + 80 mmol/day for 14 days)	327 patients	No reduction of secondary ischemia or outcome
Westermaier et al., 2010 [19]	Randomized, double-blinded	MgSO_4_ (141 ± 51 mmol – target serum level 2.0 – 2.5 mmol/l) vs. placebo	107 patients	Significant reduction of secondary infarction and ultrasonographic/angiographic vasospasm. Non-significant reduction of neurological outcome and mortality
Mees et al., 2012 [52]	Randomized, double-blinded	Nimodipine vs. nimodipine + MgSO_4_ (64 mmol/day)	1207 patients	No improvement of clinical outcome

The results are not unequivocal. The beneficial effect might depend on the dose-regimen and comedication.

Luo and coworkers compared treatment with 100 – 200 mmol MgSO_4_ to a control group without giving other calcium antagonists. The authors reported a reduced incidence of secondary brain infarction and clinical deterioration [50]. In a randomized study, Veyna and colleagues raised serum magnesium levels to 1.7 – 2.3 mmol/l for 10 days in 20 SAH patients, reported no significant side effects and a non-significant reduction of delayed vasospasm and improvement of clinical outcome compared to 20 control patients [18].

Chia and colleagues compared 23 SAH patients with historical controls. Magnesium serum concentrations were kept between 1.0 and 1.5 mmol/l. The authors found a significant reduction of angiographic vasospasm [15].

Prevedello and coworkers reported a shorter hospitalization time by high dose magnesium therapy in a non-randomized study [53]. Muroi and coworkers treated patients with a loading-dose of 16 mmol MgSO_4_ followed by a continuous infusion of 64 mmol/day. In 16 patients, magnesium infusions had to be discontinued because of severe side effects, particularly hypotension, although mean magnesium serum levels in these patients were only 1.40 mmol/l. The authors reported a trend to better clinical outcome in magnesium treated patients [21].

In a randomized, placebo-controlled multicenter study conducted by van den Bergh et al. [17], patients received a daily dose of 64 mmol MgSO_4_ for 14 days. The results of this trial were promising as magnesium reduced the risk of delayed cerebral ischemia by 34% and the risk for poor outcome by 23%. Including 283 patients, however, the study was still underpowered. In 2006, Stippler and coworkers administered 50 mmol MgSO_4_ in 38 patients and compared them to 38 matched controls in a matched cohort study [54]. They found a reduced incidence of secondary neurological deterioration and a tendency to better neurological outcome. In a south-east Asian/Australian trial, 327 patients were randomized to receive a daily dose of 80 mmol/l MgSO_4_ or placebo. The authors reported no significant benefit of magnesium-treatment regarding clinical outcome after 6 months or clinical vasospasm [20]. The results of the largest clinical trial have just recently been published by Mees and coworkers. In this European/South American multicenter study, 1,204 patients with aneurysmal SAH were enrolled and assigned to one of two study arms to either receive 64 mmol MgSO_4_ per day or serve as controls. The administration of MgSO_4_ did not improve clinical outcome assessed 3 months after SAH [52].

The results of these clinical trials regarding the efficacy of magnesium require a critical analysis. Except for the very first clinical study by Luo et al. [50], who compared magnesium treatment to a true placebo group, all patients enrolled in these trials were co-treated with nimodipine. Nimodipine, a pyrrolopyrimidine-type calcium antagonist, has been intensively tested in various clinical studies in the 1980s and 1990s. All trials but one [55] failed to find a significant improvement of patient outcome. Analyzing the pooled data for nimodipine trials, a Cochrane review still revealed an advantage of nimodipine treatment, so that this treatment is routinely used in many centers. The Cochrane article concludes, however, that the evidence for nimodipine “is not beyond any doubt” since the benefit of treatment is largely determined by one trial [55,56]. Schmid-Elsaesser et al. compared nimodipine treatment to magnesium treatment and found no marked and significant difference in the incidence of vasospasm and clinical outcome [51].

In our own placebo-controlled study 107 patients were randomized to either receive a placebo infusion or to receive a variable dose of intravenous MgSO_4_ in order to maintain serum concentrations of 2.0 – 2.5 mmol/l for 10 days or – if there was clinical, angiographic and/or ultrasonographic vasospasm – until the signs of vasospasm had disappeared or to receive a placebo infusion [19]. The patients were not treated with other calcium antagonists. The incidence of ultrasonographic/angiographic vasospasm and of secondary ischemic infarction was lower in magnesium-treated patients. The improvement of outcome and mortality did not reach the level of significance.

## Perinatal asphyxia

Magnesium has for a long time been used for the treatment of eclampsia [57]. For the treatment of eclamptic seizures, it has been found to be more effective than phenytoin and nimodipine [58,59]. Due to its reported neuroprotective effect, observational studies have been performed assessing the protective effect for the child. In very low birth weight children, Nelson et al. [60] and Schendel et al. [61] reported a lower incidence of cerebral palsy. Randomized trials for prenatal magnesium administration were called for [62]. In large randomized placebo-controlled trials, Marret et al. found a non-significant improvement of infant mortality and white-matter injury [63], Crowther et al. a significant improvement of motor function [64] and Rouse et al. a reduced rate of cerebral palsy [65]. In the MAGPIE trial, less children had neurosensory deficits assessed by questionnaires, however, without statistical significance [66] (Table 3a). The results of these clinical trials were analyzed in a meta-analysis that concluded that magnesium administration to women at risk of preterm birth is neuroprotective for the preterm fetus [67].

**Table 3:** Randomized clinical studies investigating the neuroprotective effect of magnesium administered before delivery to women at risk for preterm birth (a) and administerd to children born at term after perinatal asphyxia (b)

**Author**	**Study setup**	**Treatment arms and comedication**	**Individuals**	**Results**
**A**				
Schendel et al., 1996 [61]	Observational	Preterm administration for tocolysis. Dose variable, observational study	1097 births with very low birth-weight	Reduced risk for cerebral palsy and mental retardation
Crowther et al., 2003 [64]	Randomized	Preterm administration of 16 mmol MgSO_4_ followed by 4 mmol/h for 24 hours to mother vs. placebo	1062 women in gest. week 30 or less with birth planned within 24 hours	Lower rate of pediatric mortality and cerebral palsy in the treatment group
Marret et al., 2007 [63]	Randomized	Preterm administration of 16 mmol MgSO_4_ (4 g) single-dose over 30 minutes	573 women in gest. week 33 or less with birth planned within 24 hours	Non-significant reduction of infant mortality and white matter injury
Magpie Trial Follow-Up Collaborative Group, 2007 [66]	Randomized	Preterm administration of 16 mmol MgSO_4_ followed by 4 mmol/h for 24 hours to mother vs. placebo	3283 children born before gest. week 37	Non-significant reduction of disability after 18 months
Rouse et al., 2008 [65]	Randomized	Preterm administration of 24 mmol MgSO_4_, followed by 8 mmol/h	2241 women in gest. week 24 – 32 with birth anticipated within 24 hours	Significant reduction of cerebral palsy
**B**				
Levene et al., 1995 [68]	open	MgSO_4_ 400 mg/kg vs. 250 mg/kg	15 full-term neonates with asphyxia	400 mg/kg: Serum level 3,6 mmol/l, profound hypotension and respiratory depression
				250 mg/kg: Serum level 2.42 mmol/kg, no effect on herat rate, blood pressure and respiration
Groenendaal et al., 2002 [69]	Randomized	MgSO_4_ (250 mg/kg 30 min after birth and 125 mg/kg after 24 and 48 hours vs. placebo	22 full-term neonates with asphyxia	No effect on pathological EEG patterns
Ichiba et al., 2002 [70]	Randomized	MgSO_4_ (3 × 250 mg/kg in 24-hour intervals) vs. placebo	34 full-term neonates with asphyxia	Less pathological CT- and abnormal EEG-findings. Higher rate of oral feeding and good short-term outcome ( at 14 days of age) in magnesium-treated children
Gathwala et al., 2006 [71]	Randomized	MgSO_4_ (250 mg/kg 30 min after birth and 125 mg/kg after 24 and 48 hours vs. placebo	40 full-term neonates with asphyxia	Safe use of magnesium. No change in heart-rate, respiratory rate of blood pressure
Bhat et al., 2009 [72]	Randomized	MgSO_4_ (3 × 250 mg/kg in 24-hour intervals) vs. placebo	40 full-term neonates with asphyxia	Less neurological abnormalities and pathological CT findings
Gethwala et al., 2010 [73]	Randomized	MgSO_4_ (250 mg/kg 30 min after birth and 125 mg/kg after 24 and 48 hours vs. placebo	40 full-term neonates with asphyxia	Less EEG- and CT abnormalities and better short-term outcome in magnesium-treated children

Several authors have assessed the neuroprotective efficacy of magnesium administered after delivery to neonates with asphyxia. Levene et al. [68] and Gathwala et al. [71] assessed the safety of intravenous administrations of 250 mg/kg. Whereas Groenendaal and coworkers found no effect on pathological EEG patterns [69], Ishiba et al. reported less pathological EEG- and CT-findings, a higher rate of oral feeding and better short-term outcome [70]. Bath et al. [72] and Gathwala et al. [73] included developmental and neurological outcome measures as endpoints of their randomized placebo-controlled trials. Both groups reported a reduction of neurological deficits and abnormal CT-findings. However, both trials included only 40 patients and were underpowered to provide definitive evidence for the effectiveness of postpartum administration in perinatal asphyxia (Table 3b).

## Conclusion and future prospects

Magnesium can be beneficial in cerebral ischemia. The range of results suggests that its effect depends on the timing of treatment, route of administration, and co-medication. Different forms of ischemia are likely to require different dose regimens. Magnesium levels in serum and CSF are well controllable because they are easy to measure and follow. Compared to other calcium antagonists, magnesium may be more suitable as a component of a combination therapy [74].

### Stroke

In spite of a reproducible neuroprotective effect in experimental models of cerebral ischemia no randomized clinical trial has, to date, confirmed the neuroprotective effect in human stroke. The FAST-MAG trial, which is currently being conducted, follows a novel concept as it is the first clinical study which starts treatment before hospital admission. This trial conception considers that the delay from the onset of ischemia to the start of infusion may have been too long in the IMAGES trial to result in a neuroprotective effect. Another interesting approach for stroke therapy could be the combination of intravenous magnesium with mild systemic hypothermia as recently proposed by Zhu and Meloni after positive results in experimental studies [26,75].

### Perinatal asphyxia

The administration of magnesium to women in preterm labor has been studied in various randomized placebo-controlled trials. A meta-analysis has found a benefit for this treatment with respect to the neurological outcome for the infant [67]. The effect of magnesium adminstration to chidren born at term that suffered parinatal asphyxia is not clear. Two randomized clinical studies have reported a beneficial effect of magnesium therapy but were underpowered. To date, no larger randomized trial has been published.

### Subarachnoid hemorrhage

Similar to the prophylactic preterm magnesium administration to infants at risk to suffer perinatal hypoxia, neuroprotective drugs can be administered prior to delayed vasospasm in subarachnoid hemorrhage. Large clinical trials assessing the efficacy of magnesium in combination with nimodipine, another calcium antagonist which is, in many centers, used as a standard treatment for all patients, found no additional, synergistic effect. Pharmacologically, the co-administration of magnesium and other calcium-antagonists is the attempt to enhance neuroprotection by two similar substances. Thus, it is not surprising that magnesium and nimodipine do not exert a synergistic neuroprotective effect while specific side effects may be enhanced [21]. The results of Schmid-Elsaesser et al. showing no significant difference between nimodipine treatment and magnesium treatment, challenge the routine use of nimodipine in aneurysmal SAH [51]. The comparison of magnesium treatment to a true placebo group to prevent secondary neurological deficits after SAH showed promising results [19,50] The bioavailability may still be improved by a clinical dose-finding study. Even after continuous high-dose treatment, however, CSF levels remain markedly lower than serum levels [19] (Figure 2). For a direct – topical - dilatory effect on subarachnoid vessels, however, much higher concentrations are required [76,77]. Those are not achievable by intravenous administration without serious side effects [12]. Intrathecal administration of MgSO_4_ by a microcatheter placed directly into the subarachnoid space has been examined by Mori et al. in experimental models of SAH in rats and dogs and has demonstrated a CBF-enhancing [78] and spasmolytic effect [79]. First experiences in SAH patients suffering clinical deterioration due to cerebral vasospasm showed an attenuation of cerebral vasoconstriction [80]. Thus, an intracisternal therapy could be an interesting and promising approach for a more efficient therapy of delayed cerebral vasospasm.

## Competing interests

None of the authors has a financial or other conflicts of interest to declare.

## Authors’ contributions

TW – Design and writing of manuscript and literature work ischemia. CS – Creation of figures and literature work subarachnoid hemorrhage. EK – Literature work subarachnoid hemorrhage. NW – Literature work asphyxia experimental and clinical. FR – Literature work ischemia experimental and clinical. GHV – Design of review, drafting of the manuscript, RIE – Design of review, drafting of the manuscript. All authors read and approved the final manuscript.
